# Complete mitochondrial genome of the East Asian fish-eating bat: *Myotis ricketti* (Chiroptera, Vespertilionidae)

**DOI:** 10.1080/23802359.2019.1681316

**Published:** 2019-10-24

**Authors:** Xiangyu Hao

**Affiliations:** College of Life Sciences, Northwest A&F University, Yangling, China

**Keywords:** *Myotis ricketti*, mitochondrial genome, phylogeny

## Abstract

I report the complete mitochondrial genome (mitogenome) of the East Asian fish-eating bat (*Myotis ricketti* or *M. pilosus*), also known as the Rickett’s big footed bat. The total length of the circular *M. ricketti* mitogenome is 17,098 base pairs, containing 13 protein-coding genes (PCGs), two ribosomal RNAs, 22 transfer RNAs, and a non-coding control region (D-loop region). The gene order and organisation of this mitogenome are similar to most of other determined vertebrate mitogenomes, with the nucleotide base composition of A 34.22%, T 30.32%, C 22.80%, and G 12.66%. Besides, the mitogenomic D-loop region contains 29 copies of a tandem repeat sequence of six nucleotides (CATACG). The phylogenetic analysis indicates that *M. ricketti* is closely related to *M. macrodactylus* and *M. petax*. This study will contribute to the investigations of phylogeny and evolution for *Myotis* and its relevant taxa.

The East Asian fish-eating bat (*Myotis ricketti* or *M. pilosus*), which has large-footed and well-developed claws, is an endemic species in China, although they are also occasionally found in Laos (Ma, Jones, et al. 2003). Previous studies found that *Myotis ricketti* is a piscivorous bat species through the faecal analysis (Ma, Jones, et al. 2003). This fish-eating species can utilise echolocation to detect water ripples produced by fish on the surface of water (Ma, Zhang, et al. 2003), making it an excellent model for studying the co-evolution between bats and fishes.

The bat was sampled at Yanziyan Mountain (Huizhou, Guangdong Province, China) (g114.394690, 22.951571) on June 30, 2018. Genetic material of this bat (WHU-2018-SB140436) is stored in the Herbarium of Wuhan University. The genomic DNA was isolated from the liver tissue using the Universal DNA Purification Kit (TIANGEN BIOTCECH, Beijing). The complete mitochondrial genome of *M. ricketti* (GenBank accession number MN245054) was amplified through PCRs using 16 pairs of newly designed primers according to the mitogenome sequences of other determined *Myotis* species (Nam et al. 2015; Kim et al. 2017). The genome is 17,098 bp in size and contains 13 protein-coding genes (PCGs) (*Nd1*, *ND2*, *Cox1*, *Cox2*, *ATP8*, *ATP6*, *Cox3*, *ND3*, *ND4L*, *ND4*, *ND5*, *ND6*, and *Cytb*), two rRNA genes (*12S rRNA* and *16S rRNA*), 22 tRNA genes, and one non-coding control region (D-loop region) (Wilkinson et al. 1997), with its gene order and organisation similar to those of other mammals (Yoon et al. 2015). The base contents of A, T, C, and G are 34.22%, 30.32%, 22.80%, and 12.66%, respectively, showing a relatively strong AT bias. The total length of the 13 PCGs is 11,376 bp. All PCGs are encoded in H-strand except for *ND6*, which is encoded in L-strand. All PCGs begin with the start codon ATG except *ND2* (using ATT) and terminate with stop codons TAA, TAN, TNN, or AGA.

Like other mammals, the 22 tRNA genes totalling of 1512 bp comprise three major clusters: IQM (*tRNA^Ile^*, *tRNA^Gln^*, and *tRNA^Met^*), WANCY (*tRNA^Trp^*, *tRNA^Ala^*, *tRNA^Asn^*, *tRNA^Cys^*, and *tRNA^Tyr^*) and HSL (*tRNA^His^*, *tRNA^Ser(AGY)^*, and *tRNA^Leu(CUN)^*), and the replication origin region (O_L_) is located between *tRNA^Asn^* and *tRNA^Cys^*. Each of the 22 tRNAs could be folded into a cloverleaf secondary structure, with the exception of *tRNA^Ser(AGY)^* lacking the ‘DHU’ arm. The *12S rRNA* and *16S rRNA* are 961 bp and 1566 bp long, respectively, and separate the *tRNA^Phe^*, *tRNA^Val^*, and *tRNA^Leu(UUR)^* in turn. In addition, the total length of D-loop region is 1645 bp, containing 29 tandem repeats (CATACG).

The phylogenetic relationships of 26 *Myotis* species were reconstructed with neighbour-joining (NJ) and maximum-likelihood (ML) methods based on the alignment of nucleotide sequences of 13 PCGs, with two vesper bat species (*Plecotus auritus* and *P. macrobullaris*) as the outgroups (Figure 1). The results showed that both NJ and ML trees contain two strongly supported clades (clade 1 and clade 2) (Figure 1), which were also reported earlier (Chung et al. 2018). In addition, *M. ricketti* is most closely related to *M. macrodactylus* and *M. petax*.

**Figure 1.**
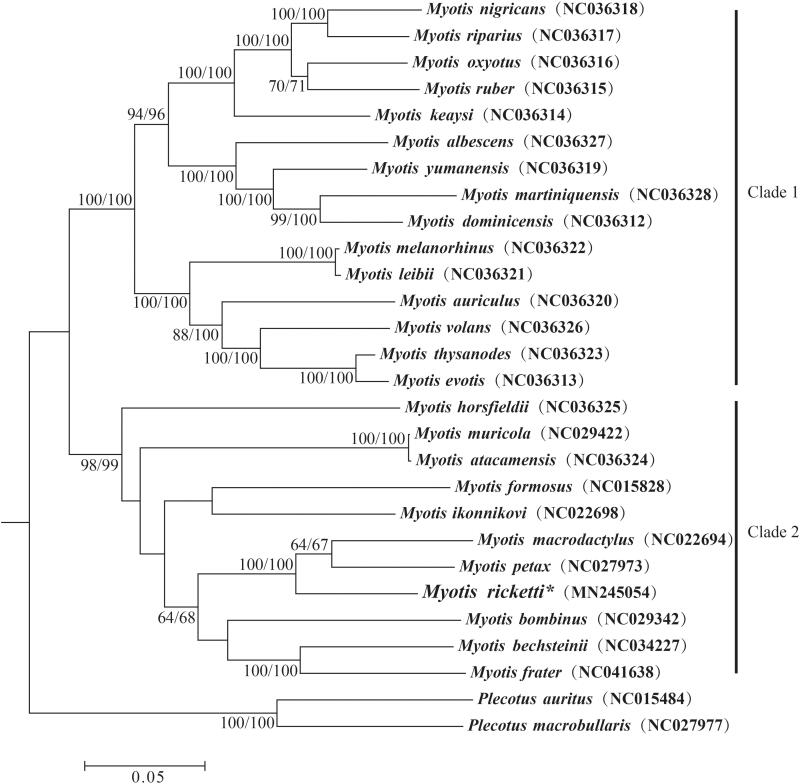
The phylogenetic relationship of *Myotis ricketti* and its related 25 species inferred from neighbour-joining and maximum-likelihood analyses. Numbers at the nodes are the ML/NJ bootstrap values from both analyses.
